# A new global agenda for nutrition and health: the importance of agriculture and food systems

**DOI:** 10.2471/BLT.15.164509

**Published:** 2016-02-03

**Authors:** Andrew D Jones, Gebisa Ejeta

**Affiliations:** a School of Public Health, University of Michigan, Ann Arbor, United States of America (USA).; b Center for Global Food Security, Purdue University, 203 S Martin Jischke Drive (Mann Hall Room 105D), West Lafayette, Indiana, 47907-1971, USA.

The second of the new sustainable development goals commits Member States to “end hunger, achieve food security and improve nutrition, and promote sustainable agriculture”. Unifying the aspirations of the nutrition and sustainable agriculture communities into a single statement presents a unique opportunity to align the goals of these sectors in a common and even more ambitious cause.

A productive, diverse, ecologically and socially sustainable agricultural sector has long been recognized as crucial for shaping healthy diets and improving human nutrition. More than three-quarters of a century ago, the League of Nations recognized the importance of agricultural adaptation for dietary diversification, noting that changes in production decisions that supported more protective foods (i.e. fruits and vegetables), could lead to nutritional benefits.^1^ Observers today continue to call attention to the importance of food systems for shaping human health and nutrition.^2^^,^^3^ That our systems of food production should be designed to meet recommendations for healthy diets seems obvious. Yet, the goals of agriculture and nutrition have often diverged.

Following the Second World War, increasing food production was seen as fundamental to fighting hunger, reducing social inequities and lifting families out of poverty. Investments in agricultural research to develop high-yielding varieties of wheat and rice helped to double cereal yields in Asia and Latin America. This Green Revolution averted global food shortages and saved millions of lives. Part of its legacy however, has been a persistent emphasis on expanding production of a select few staple grains. This calories-first inheritance has limited the contribution of agriculture to meeting most national dietary recommendations that emphasize consumption of fruits, vegetables and pulses, as well as cereals. Reshaping modern agriculture and food systems to be more nutrition-sensitive has been only a secondary concern of most nutrition programmes and policies. Instead, efforts to strengthen primary health care, and reduce deficiencies of specific micronutrients through supplementation and fortification have been favoured. These efforts, and those of agriculture to generate an abundance of affordable food, have been enormously important for reducing both severe acute and chronic malnutrition. However, the global landscape of malnutrition has shifted in recent decades.

Countries that have made impressive strides in reducing undernutrition (e.g. Brazil, China and Chile) have also experienced rapid increases in the prevalence of obesity and related chronic disease.^4^ Unfortunately, many countries now face a double burden of undernutrition and obesity as economic development, changing diet patterns, sedentary lifestyles and urban migration have outpaced efforts to develop infrastructure, strengthen institutions and expand provision of health services.^5^ More than ever, integrative solutions are needed that are able to combat malnutrition on multiple fronts, across the spectrum from deficiencies of energy and micronutrients to overconsumption, obesity and related diseases. The food and agriculture sectors are uniquely positioned to provide such solutions.

The complexity of the diet-health nexus and an emphasis on the health impacts of specific dietary compounds rather than foods has made it difficult to communicate a consistent and clear message to guide the goals of food production. Yet, an increasing number of academics, health professionals and practitioners advocate that foods, rather than nutrients, should serve as the basis for dietary recommendations.^6^ From a food-based perspective, there is broad and consistent scientific evidence to support a simple dietary guideline for optimizing health: eat a variety of real foods; mostly plants.^7^ Agriculture is essential to meeting this simple directive. Yet, in many ways, our current systems of agriculture have not been designed for the production of a diversity of nutrient-dense foods.

Just three food crops – rice, maize and wheat – provide nearly two-thirds of global dietary energy intake. The global supply of pulses, fruits and vegetables, the primary sources of diversity in most diets, is insufficient to meet recommended population-level intakes.^8^ At the same time, agriculture has increasingly become an engine not for producing food, but for generating animal feed, biofuels and industrial ingredients for processed food products (e.g. sugar-sweetened beverages, ready-to-eat meals and snacks).^9^

If the goals of agriculture are to be aligned with our aspirations for healthy diets, diversity must be prioritized alongside the critical goal of enhancing staple crop productivity. New investments in agricultural research, and perhaps more aptly, food systems research, are needed to develop technologies for production of pulses, fruits and vegetables at lower cost. Parallel efforts to strengthen the functioning of markets and adapt food value chains must accompany enhanced production efficiencies. These efforts are important for supporting stable incomes for farmers expanding the production of vegetables, pulses and fruits, while ensuring that the nutritional quality of these foods is not diminished after harvest.

These investments and actions are only sensible if coupled with parallel investments elsewhere in the food system to increase demand for nutrient-dense foods.^10^ Evidence-based behaviour-change strategies are needed that reflect the enormously successful commercial marketing approaches used by the food and beverage industry to promote highly processed foods and beverages. Cultivating food environments that promote health is also essential. In some contexts, regulatory approaches may be appropriate – for example, taxation of sugar-sweetened beverages, mandatory front-of-pack labels on packaged food products or limits on marketing of certain foods to children.^11^ However, influencing behavioural cues by subtly altering the convenience and attractiveness of health-promoting pulses, vegetables and fruits in schools, markets, workplaces and in homes may also be effective without restricting choices.^12^ The public health and nutrition communities also need to more fully integrate food systems approaches into the training of health professionals, into the goals and designs of their respective programmes, as well as into policy advocacy efforts, as one strategy in an ever-evolving toolkit for addressing malnutrition.

The complex nutritional challenges that we now face – reflected in the double burden of undernutrition and obesity– are daunting, but not insurmountable. These challenges defy solutions that rely on conventional sectoral approaches, but in doing so, invite new thinking that could afford multiple benefits. For example, there is enormous unrealized market potential in the production of pulses, fruits, vegetables and ancient grains that could contribute to new livelihood opportunities for millions of smallholder farmers.^13^ These same crops are essential for preventing undernutrition, obesity and diet-related disease that together are contributing to increase in health-related costs and lost productivity.

Therefore, with consonant objectives, global health and food systems could help to restore the vitality of economies globally. Realigning the goals of agriculture to address human health by meeting dietary guidelines is only the most conspicuous of many possible pathways through which food systems can be leveraged to affect human health and nutrition. Food systems, for example, also have potential to affect food safety, exposure to infectious illness, food prices, household incomes and women’s access to productive resources – all of which are key mediators of nutrition and health.^14^ Since agriculture contributes up to one-third of global greenhouse gas emissions, new and improved climate-smart food production and processing systems could have a particularly positive impact on both the environment and the many human health and nutrition outcomes that otherwise would be negatively affected by extreme weather.^15^

It is our hope that the agriculture, nutrition and health communities can leverage the potential for shared solutions across sectors to confront the challenge of improving nutrition and health and ending global hunger while protecting the global ecosystem services on which food production depends.
